# Partial mtDNA sequencing data of vulnerable *Cephalopachus bancanus* from the Malaysian Borneo

**DOI:** 10.1016/j.dib.2019.104133

**Published:** 2019-06-25

**Authors:** Muhamad Aidil Zahidin, Norehan Abd Jalil, Nur Mukminah Naharuddin, Mohd Ridwan Abd Rahman, Millawati Gani, Mohd Tajuddin Abdullah

**Affiliations:** a Institute of Tropical Biodiversity and Sustainable Development, Universiti Malaysia Terengganu, 21030 Kuala Nerus, Malaysia; b Faculty of Resource Science and Technology, Universiti Malaysia Sarawak, 94300 Kota Samarahan, Malaysia; c Faculty of Science, Universiti Putra Malaysia, 43400 UPM Serdang, Malaysia; d Center for Pre-University Study, Universiti Malaysia Sarawak, 94300 Kota Samarahan, Malaysia; e National Wildlife Forensic Laboratory, Department of Wildlife and National Park Peninsular Malaysia, KM 10, Jalan Cheras, 56100 Kuala Lumpur, Malaysia; f School of Marine and Environmental Sciences, Universiti Malaysia Terengganu, 21030 Kuala Nerus, Malaysia

**Keywords:** Cytochrome b gene, Population genetics, Primate, Tarsier, Borneo

## Abstract

Tarsier is an endangered nocturnal primate in the family Tarsiidae and is an endemic to Sundaic islands of Philippine (*Carlito syrichta*), Sulawesi (*Tarsius* tarsier-complex) and Borneo (*Cephalopachus bancanus*). Recent records indicated that most molecular studies were done on the Eastern Tarsier and little information for the other group of tarsiers. Here, we present a partial cytochrome b data set of *C. bancanus* in Sarawak, Malaysian Borneo. Standard mist nets were deployed at strategic locations in various habitat types. A total of 18 individuals were caught, measured and weighed. Approximately, 2 × 2 mm of tissue samples were taken and preserved in molecular grade alcohol. Out of 18, only 11 samples were screened with partial mtDNA (cytochrome *b*) and the DNA sequences were registered in the GenBank (accession numbers: KY794797-KY794807). Phylogenetic trees were constructed with 20 additional mtDNA sequences downloaded from GenBank. The data are valuable for the management authorities to regulate the type of management units for the metapopulation to sustain population genetics integrity of tarsiers in the range countries across the Sunda Shelf.

**Table:** Specifications table

Subject area	Biology
More specific subject area	Molecular Evolution
Type of data	Cytochrome *b* partial data are presented as in Table 1, Table 2, Table 3, Table 4, Table 5, Table 6, Fig. 1, Fig. 2, Fig. 3 and Supplementary Tables 1–3.
How data was acquired	Data were acquired by extracting and amplifying, purifying (Promega Wizard SV Gel and PCR Clean-Up System (Promega Co.), and sequencing (First Base Laboratories Malaysia) the target mtDNA region and analysed using Sequencher 5.4 (https://genecodes.com), ClustalW2 MUSCLE (https://www.ebi.ac.uk), MEGA 7 and DnaSP software.
Data format	Raw and analysed data
Experimental factors	The sequence alignments were trimmed and filtered
Experimental features	Phylogenetic analyses of partial cytochrome *b*
Data source location	Sarawak, Malaysian Borneo and GenBank
Data accessibility	GenBank with accession number KY794797-KY794807 (https://www.ncbi.nlm.nih.gov/nuccore/?term=Cephalopachus+bancanus+bancanus+isolate)
Related research article	M.T. Abdullah, Mammalian Evolution and Biogeography (Evolusi dan Biogeografi Mammalia), Universiti Malaysia Terengganu, Kuala Nerus 2016.

**Table:** 

**Value of the Data** • The data are valuable for the management authorities to determine the type of management units for the metapopulations to maintain the integrity of population genetics in their ranges across the Sunda Shelf. • The data can be used as baseline information for future studies on genetic and molecular ecology that can be used as a flagship model to test the “Out of Sunda” theory and elucidating the history of prehistoric humans and primates migration waves in Southeast Asia. • The data allow other researchers focusing on this population to start the genome-wide analysis.

## 1 Data

Tarsiers are a vulnerable primate group [1] in family Tarsiidae that can be found on Southeast Asia Islands; Sundaic islands of Philippine (*Carlito syrichta*), Sulawesi and surrounding islands (*Tarsius* tarsier-complex) and Borneo (*Cephalopachus bancanus*) [2]. Western Tarsier *Cephalopachus bancanus bancanus* can be found in Malaysian Borneo and is listed as protected and totally protected species in the Malaysia's Wildlife Conservation Act (WCA) 2010 and Sarawak's Wildlife Protection Ordinance (WLPO) 1998 respectively. The molecular research interest on this endemic species is due to the availability of recent information related to taxonomy and evolutionary relationship of tarsiers since the expansion of fauna and prehistoric human into Southeast Asia [2], [3].

**Fig. 1:**
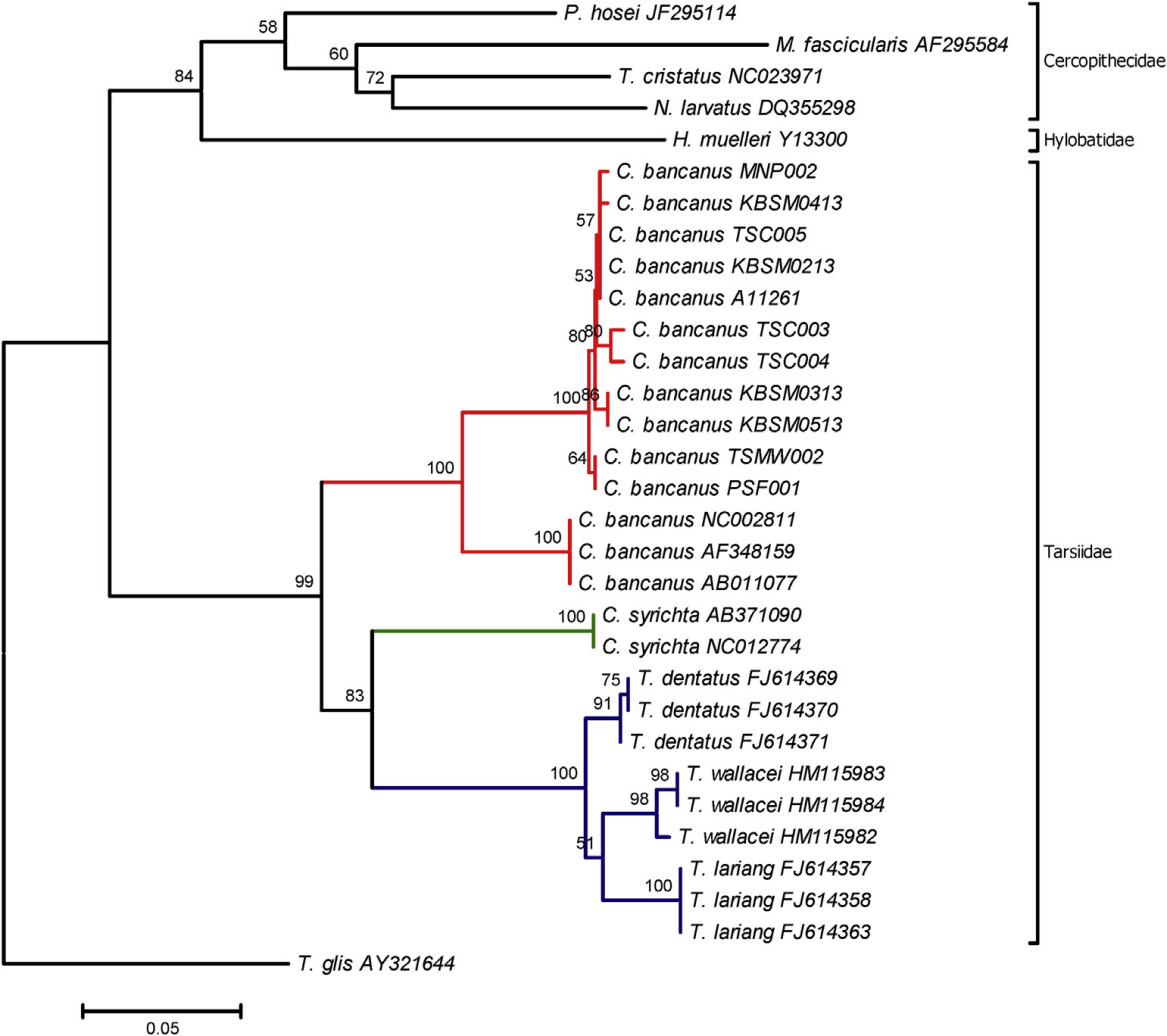
The evolutionary history was inferred using the Neighbor-Joining tree method. The optimal tree with the sum of branch length = 1.16079630 is shown. The percentage of replicate trees in which the associated taxa clustered together in the bootstrap test (1,000 replicates; more than 50%) is shown above the branch. The institutional codes are listed in Table 2, Table 4.

**Fig. 2:**
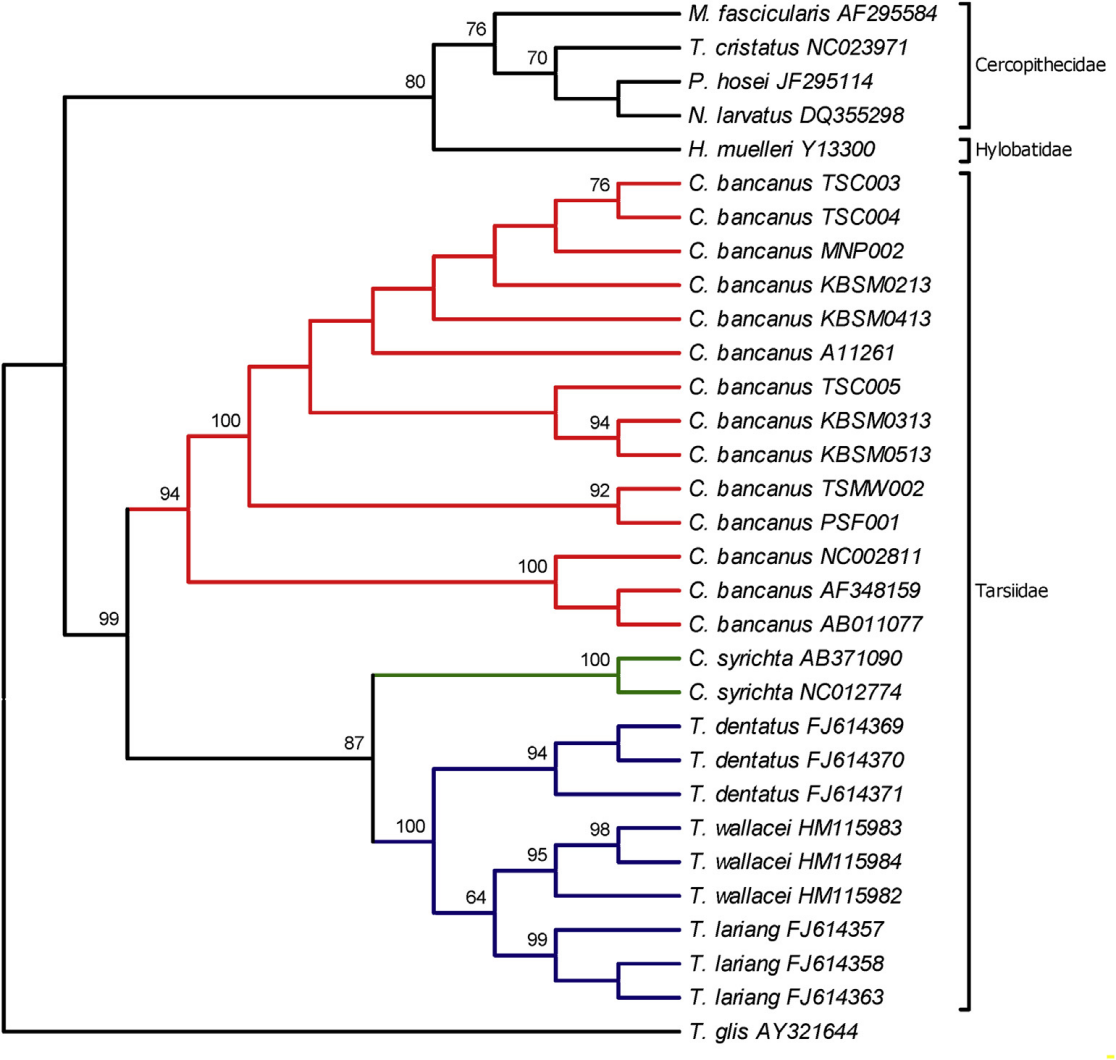
The evolutionary history was inferred using the Maximum Parsimony method. The tree was obtained using the Subtree-Pruning-Regrafting (SPR) algorithm which the initial trees were obtained by the random addition of sequences. The consistency index is 0.819864 and the composite index is 0.478254 for all sites and parsimony-informative sites. The percentage of replicate trees in which the associated taxa clustered together in the bootstrap test (1,000 replicates; more than 50%) is shown above the branch. The institutional codes are listed in Table 2, Table 4.

**Fig. 3:**
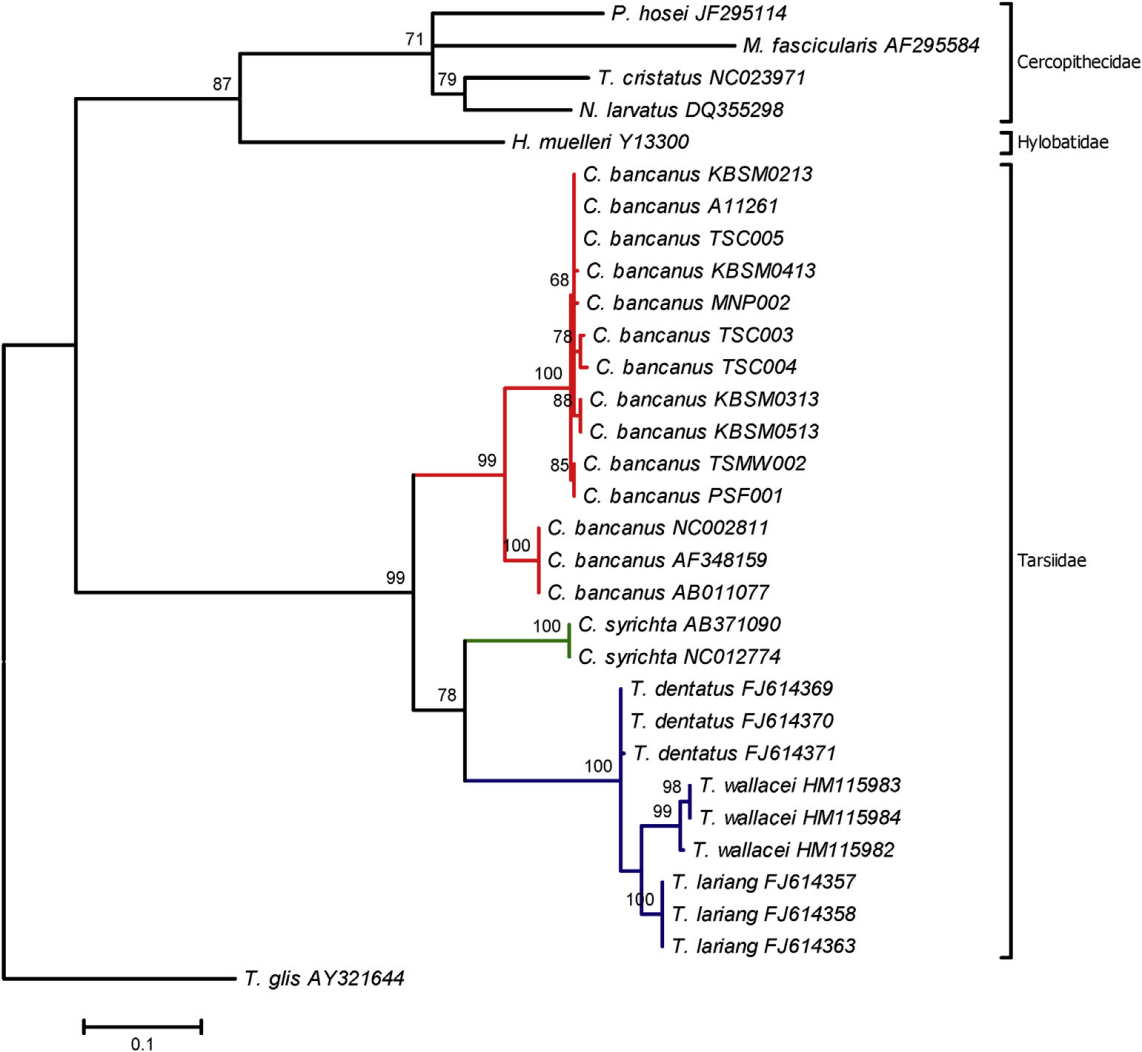
The evolutionary history was inferred by using the Maximum Likelihood method based on the Hasegawa-Kihino-Yano (HKY + G + I) model and the tree with the highest log likelihood (−2336.6352) is shown. The percentage of replicate trees in which the associated taxa clustered together in the bootstrap test (1,000 replicates; more than 50%) is shown above the branch. The institutional codes are listed in Table 2, Table 4.

This dataset contains genetic phylogenetic information of *C. bancanus* from Malaysian Borneo. Table 1 shows a list of field sampling conducted in Sarawak, Borneo. Field number, standard morphological measurements, weight and sex of each individual were recorded as in Table 2. A set of partial primers of Cytochrome *b*, DNA master mixture profile and PCR profile were tabulated as in Table 3 and Supplementary Tables 1 and 2 respectively [4]. Additional 20 mtDNA sequences derived from the GenBank [5], [6], [7], [8], [9], [10], [11], [12], [13], [14], [15] were used and tabulated in Table 4. The sequence variations, frequency distribution haplotypes and pairwise distance of tarsier were identified as in Table 5, Table 6 and Supplementary Table 3. The evolutionary relationships of taxa were inferred using the Neighbor-Joining, Maximum Parsimony and Maximum Likelihood methods are shown as in Fig. 1, Fig. 2, Fig. 3.

**Table 1:** Field sampling conducted in Sarawak, Borneo.

	Division	Sampling site	Coordinate
1	Betong	Maludam National Park	1.5271° N, 111.1414° E
2	Kota Samarahan	Universiti Malaysia Sarawak	1.4649° N, 110.4269° E
3	Kuching City	Cermat Ceria Forest	1.^o^ 24′01.6″ N, 111° 23′54.0″ E
4	Kuching City	Durafarm Plantaion	1° 23′50.63697″ N, 111° 50.59624″ E
5	Kuching City	Kampung Barieng	1° 25′0″ N, 110° 0′9″ E
6	Kuching City	Kubah National Park	1.6128° N, 110.1969° E
7	Kuching City	Matang Wildlife Centre	1.6166° N, 110.1582° E

**Table 2:** Taxonomic measurements of captured *C. bancanus* with their registered accession number in the GenBank.

	Field no.	Species	Measurements (mm)										Wt (g)	Sex	Note	Accession Number
			E	HF	TV	HB	TL	RH	LH	RF	LF	Chest				
1	TSKN P001	*Cephalopachus bancanus*	40.23		214	130	344	48.04	46.96	42.95	42.53	110	68	M	Kubah National Park	
2	TSKNP 002	*C. bancanus*	27.71	40.79	206	136	342	46.03	45.67	40.79	40.71	126.93	150	F	Kubah National Park	
3	TSC 002	*C. bancanus*													UNIMAS	
4	TSC 003	*C. bancanus*	27.60		191	149	340	40.64	40.32	36.9	37.3		105	F	UNIMAS	KY794803
5	TSC 004	*C. bancanus*	38.66		225	143	368	49.3	50	39.1	39		110	F	UNIMAS	KY794804
6	TSC 005	*C. bancanus*	28.00	65.64	24.6	117	423	45	45	35	35	67		M	UNIMAS	KY794805
7	TSMW 001	*C. bancanus*	30.00		138			45	47	40	40	115	110	M	Matang Wildlife Centre	
8	TSMW 002	*C. bancanus*	28.00	67.00	216	119		47	47	37	37	92		M	Matang Wildlife Centre	KY794807
9	MNP 001	*C. bancanus*	23.00	72.00	241								121	M	Maludam National Park	
10	MNP 002	*C. bancanus*	21.80	73.99	200								124	M	Maludam National Park	KY794806
11	PSF 001	*C. bancanus*	31.00		266	140	406						115	M	Cermat Ceria Forest	KY794801
12	PSF 002	*C. bancanus*	25.23		220	154	374						130	M	Cermat Ceria Forest	
13	KBSM 1302	*C. bancanus*	31.00	76.00	225	132	357						119	F	Kampung Barieng	KY794797
14	KBSM 1303	*C. bancanus*	22.00	71.00	225	150	375						125	M	Kampung Barieng	KY794798
15	KBSM 1304	*C. bancanus*	30.00	70.00	219	140	359						108	F	Kampung Barieng	KY794799
16	KBSM 1305	*C. bancanus*	25.00	74.00	225	150	375						123	M	Kampung Barieng	KY794800
17	A08897	*C. bancanus*	21.07	64.62	210	133	343						133	M	Durafarm Plantation	
18	A11251	*C. bancanus*	20.05	76.00	230	141	371							M	Durafarm Plantation	KY794802

E- Ear length, HF- Hind foot length, T- Tail length, HB- Height body length, TL- Total length, RH- Right hand length, LH- Left hand length, RF- Right foot length, LF- Left foot length.

M- Male, F- Female, UNIMAS- Universiti Malaysia Sarawak.

**Table 3:** Primer used for PCR amplification [4].

Primer	Primer sequences (5′-3′)	Size (bp)
Glud-GL (F)	5′- TGACCTGARAACCAYCGTTG -3′	500
CB2H (R)	5′- CCTTCAGAATGATATTTGTCCTCA -3′	500

**Table 4:** Additional 20 mtDNA sequences used in this study.

	Scientific name	Common name	Accession Number	Author
1	*Cephalopachus bancanus*	Western tarsier	NC002811	[5]
2	*C. bancanus*	Western tarsier	AF348159	[5]
3	*C. bancanus*	Western tarsier	AB011077	[6]
4	*Carlito syrichta*	Philippine's tarsier	AB371090	[7]
5	*C. syrichta*	Philippine's tarsier	NC012774	[7]
6	*Tarsius wallacei*	Eastern tarsier	HM115983	[8]
7	*T. wallacei*	Eastern tarsier	HM115984	[8]
8	*T. wallacei*	Eastern tarsier	HM115982	[8]
9	*T. lariang*	Eastern tarsier	FJ614357	[9]
10	*T. lariang*	Eastern tarsier	FJ614358	[9]
11	*T. lariang*	Eastern tarsier	FJ614363	[9]
12	*T. dentatus*	Eastern tarsier	FJ614369	[9]
13	*T. dentatus*	Eastern tarsier	FJ614370	[9]
14	*T. dentatus*	Eastern tarsier	FJ614371	[9]
15	*Hylobates muelleri*	Bornean gibbon	Y13300	[10]
16	*Macaca fascicularis*	Long-tailed macaque	AF295584	[11]
17	*Trachypithecus cristatus*	Silvered-leaf monkey	NC023971	[12]
18	*Nasalis larvatus*	Proboscis monkey	DQ355298	[13]
19	*Presbytis hosei*	Hose's langur	JF295114	[14]
20	*Tupaia glis*	Common treeshrew	AY321644	[15]

**Table 5:** Sequence variation of Western Tarsier.

Indices	Partial Cyt *b*
Base pair	375 bp
Conserved site	366
Variable site	9
Parsimony-informative site	5
Singleton	4
Nucleotide composition (%) C	26.40
T	30.20
A	27.20
G	16.20
Overall mean distance	0.007

**Table 6:** Frequency distribution of the partial Cyt *b* haplotypes.

Haplotype	n	Sample	Frequency
Hap 1	1	*C. bancanus* TSC003	0.091
Hap 2	1	*C. bancanus* TSC004	0.091
Hap 3	3	*C. bancanus* TSC005, *C. bancanus* KBSM0213, *C. bancanus* A11261	0.273
Hap 4	2	*C. bancanus* TSMW002, *C. bancanus* PSF001	0.182
Hap 5	1	*C. bancanus* MNP002	0.091
Hap 6	2	*C. bancanus* KBSM0313, *C.bancanus* KBSM0513	0.182
Hap 7	1	*C. bancanus* KBSM0413	0.091

## 2 Experimental design, materials and methods

### 2.1 Sample Collection

Field sampling was conducted at the southern part of Sarawak; Kubah National Park, Matang Wildlife Centre, Universiti Malaysia Sarawak (UNIMAS), Maludam National Park, Cermat Ceria Forest, Kampung Barieng and Durafarm Plantation (Table 1). The samplings were assisted by the field assistants from the Institute of Biodiversity and Environmental Conservation (IBEC), UNIMAS. A total of ten mist nets were deployed at strategic locations with high vegetation, trees with small trunk diameter and near to the stream or water bodies [16], [17], [18], [19], [20]. A total of 18 individuals were captured, identified, sexed, measured and weighed (Table 2) [18], [19], [20], [21]. Each was tranquilised using Zoletil 100 mg solution. Approximately, 2 × 2 mm-thick tissues samples were taken and preserved in molecular grade alcohol.

### 2.2 DNA extraction, amplification, purification and sequencing

The DNA samples were extracted using cetyl-tri-methyl ammonium bromide (CTAB) protocol [22] and polymerase chain reaction (PCR) amplified using a set of cytochrome *b* partial primers [4]. The amplified products were purified using Promega Wizard SV Gel and PCR Clean-Up System (Promega Co.) and subjected to cycle sequencing at the First Base Laboratories Malaysia. The *C. bancanus* sequences were registered in the GenBank (accession numbers: KY794797-KY794807) (Table 2).

### 2.3 Sequence analysis

The nucleotide sequences were visualized and read using Sequencher 5.4 (https://genecodes.com). The sequences were matched and aligned with 20 additional mtDNA sequences (Table 4) [5], [6], [7], [8], [9], [10], [11], [12], [13], [14], [15] using ClustalW2 MUSCLE (Multiple Sequence Comparison by Log-Expectation) (https://www.ebi.ac.uk). The nucleotide composition and haplotype frequency were performed in Molecular Evolutionary Genetics Analysis (MEGA) 7 [23] and DnaSP [24]. The evolutionary divergence between sequences (Supplementary Table 3) was estimated in MEGA 7 by using the p-distance model where all positions containing gaps and missing data were eliminated. Kimura 2-parameter method was used to compute the Neighbor-Joining tree (Fig. 1). The evolutionary history of Maximum Parsimony was shown in Fig. 2. The tree was obtained using the Subtree-Pruning-Regrafting (SPR) algorithm which the initial trees were obtained by the random addition of sequences. Meanwhile, the evolutionary history of Maximum Likelihood was performed using the Hasegawa-Kishino-Yano (HKY + G + I) method (Fig. 3). The best model was chosen based on the Akaike Information Criterion (AIC; 4776.487) value and the lowest Bayesian Information Criterion (BIC; 5254.204) score.
