# Supplementary material for: Partial mtDNA sequencing data of vulnerable Cephalopachus bancanus from the Malaysian Borneo
Source: Data Brief. 2019 Jun 25;25:104133. doi: 10.1016/j.dib.2019.104133 (PMC6613094; doi:10.1016/j.dib.2019.104133)
Supplement: Multimedia component 1 [file mmc1.docx]

**Supplementary Table 1**

Master mixture profile of 25 µl reaction volume [4].

| **Reagents** | **Reaction mixtures (µl)** |
| --- | --- |
| 10 X PCR reaction buffer | 2.5 |
| 50 mM MgCl_2_ | 1.5 |
| dNTP | 0.5 |
| Forward primer (Glud-GL) | 1.0 |
| Reverse primer (CB2H) | 1.0 |
| ddH_2_O | 15.3 |
| DNA template | 2.0 |
| Taq Polymerase | 0.2 |
| **Total** | **25** |

**Supplementary Table 2**

Polymerase Chain Reaction (PCR) profile [4].

| **Phase** | **Temperature (^o^C)** | **Time** | **Number of cycle** |
| --- | --- | --- | --- |
| Pre-denaturation | 93 | 2 min |  |
| Post-denaturation | 93 | 1 min |  |
| Annealing | 50.2 | 1 min | 30 |
| Extension | 72 | 2 min |  |
| Post-extension | 72 | 5 min |  |
| Soak | 4 | ∞ |  |
